# Thrombotic thrombocytopenic purpura developed after pegylated interferon treatment for hepatitis B infection

**DOI:** 10.1186/s12882-022-03034-9

**Published:** 2022-12-13

**Authors:** Shuqin Mei, Yun Feng, Linlin Cui, Jing Chen, Zhiguo Mao, Xuezhi Zhao, Changlin Mei, Yixin Qian

**Affiliations:** 1 Department of Nephrology, Second Affiliated Hospital of Naval Medical University, Shanghai, 200003 China; 2 Department of Hepatic Surgery, Fudan University Shanghai Cancer Center, Shanghai Medical College, Fudan University, Shanghai, 200032 China

**Keywords:** Thrombotic microangiopathy, Thrombotic thrombocytopenic purpura, Hepatitis B, Pegylated interferon, COVID-19 vaccination

## Abstract

**Background:**

Thrombotic thrombocytopenic purpura (TTP) is a rare and life-threatening thrombotic microangiopathy characterized by microangiopathic hemolytic anemia, severe thrombocytopenia, and organ ischemia. It is related to severe deficiency in ADAMTS13, which is usually acquired via ADAMTS13 autoantibodies or inherited via mutations of the *ADAMTS13* gene. The etiology of acquired TTP including HIV infection, pregnancy, autoimmune disease, organ transplantation, drugs, malignancy and so on. Here, we firstly reported a patient diagnosed as acquired TTP after pegylated interferon therapy for hepatitis B and COVID-19 vaccination.

**Case presentation:**

A 36-year-old male attended to our unit with a five-day history of intermittent hematuria and progressive fatigue on January 5th, 2022. He had a 13 years history of hepatitis B infection and undergone pegylated interferon treatment (which was paused for two months because of COVID-19 vaccination) for nearly 3 years. Laboratory evaluation revealed a haemoglobin level of 61 g/L, platelet count of 11 × 10^9^/L, lactate dehydrogenase 2133 U/L. The direct and indirect Coombs test were both negative. On a peripheral blood smear, there were about 18.8% schistocytes. Meanwhile, the results of ADAMTS 13 activity and antibody were < 5% and 181.34 ng/ml (131.25–646.5), respectively

**Conclusion:**

This case firstly reported the rare complication of TTP after pegylated interferon treatment for hepatitis B and COVID-19 vaccine injection. This unique sign warrants more attention as an early cue of diagnosis of TTP and be aware of the rarity adverse effect of interferon therapy and COVID-19 vaccination.

## Background

The last two decades have  been marked by the connection between an old disease, the thrombotic thrombocytopenic purpura (TTP), and a protein ADAMTS13 (the 13th member of the ADAMTS protein family). For now, TTP is well established as a rare hematologic disease with an extremely low average annual prevalence. It mostly occurs during adulthood and about twofold more frequent in female and its clinical course is characterized by a relapsing tendency. Severe ADAMTS13 deficiency is the only causing factor for TTP identified so far, which is due to the presence of anti- ADAMTS13 autoantibodies (acquired TTP) or *ADAMTS13* gene mutation (inherited TTP). Plasma exchange, with or without steroids, anti-CD20 monoclonal antibody rituximab and caplacizumab are showing promising in the management of TTP.

Interferon therapies is not uncommon in the treatment of several diseases and interferon related thrombotic microangiopathy has been reported during past years. However, there is little known about the TTP induced by interferon treatment for hepatitis B infection, not to mention combination with the COVID-19 vaccination.

With regard to the importance of a differential diagnosis of thrombotic microangiopathy disorders, we firstly present a case of a young man with more than 2 years of interferon treatment and two times COVID-19 vaccination history who was finally further confirmed by genetic analysis as acquired TTP.

## Case presentations

A 36-year-old Asian male attended to our unit with a five-day history of intermittent hematuria and progressive fatigue on January 5th, 2022. He had a 13 years history of hepatitis B infection and was treated with oral lamivudine or adefovir disoproxil. Since February 16, 2019, he regularly undergone peroxin (peginterferon-2 α) 180 ug/week (which was paused for two months because of the two vaccinations for COVID-19 in May and June, 2021) and tenofovir disoproxil fumarate tablet 300 mg/day for antiviral therapy. And the latest interferon therapy was 5 days before he came to our unit. During the interferon treatment, the patient’s blood pressure and blood tests were monitored every 3 months, including complete blood count (CBC), liver enzymes and renal function, with no abnormal results.

**Fig. 1:**
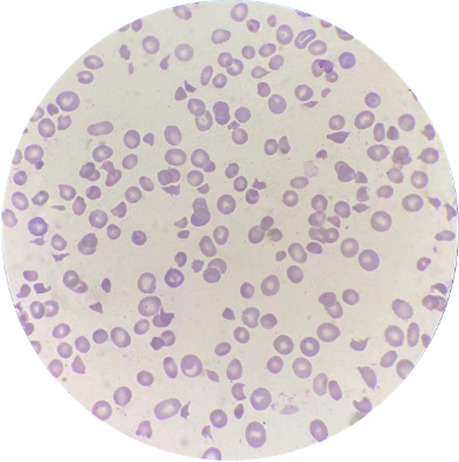
Patient’s peripheral blood smear showing schistocytes

On physical examination, he was pale, not alert and oriented to time, place. His temperature was 36.6℃, pulse was 86 bpm, blood pressure was 112/80 mmHg, and respiratory rate was 23 bpm on room air. Examination of the skin and mucosa did not show any purpura, or active bleeding, just showed sporadic petechiae on both lower extremities. Laboratory evaluation revealed a haemoglobin (Hb) level of 61 g/L (normal range, 115–150), hematocrit 18.6% (normal range, 35–45), reticulocytosis of 15.29% (0.5%–1.5%), platelet count of 11 × 10^9^/L (normal range, 125 to 350). Prothrombin, activated partial thromboplastin time and fibrinogen level were normal. D-Dimer was elevated (2.43 μg/ml; normal range < 0.55). Biochemical tests also showed the following were abnormal: lactate dehydrogenase (LDH) 2133 U/L (120–246), total bilirubin 47 umol/L (3–22), unconjugated bilirubin 47 umol/L (2–19), while, blood urea nitrogen 6 mmol/L (2.6–6.1) and creatinine 94 umol/L (57–111) were normal. Urinalysis revealed protein 1 + , blood 2 + with red cell count of 3.4/ul. The direct and indirect Coombs test were both negative. On a peripheral blood smear, there were about 18.8% schistocytes. Based on the presence of fever (38.6℃, 2 h later after admission), lethargy concomitantly with thrombocytopenia and microangiopathic hemolytic anemia, a presumptive diagnosis of autoimmune TTP was made. After obtaining blood sample for ADAMTS 13 activity test, a central venous catheter was placed and daily therapeutic single volume plasma exchange (PE) begun along with low dose of methylprednisolone (40 mg/d) at the following day. For hepatitis treatment, we paused the peginterferon-2 α therapy when he attended to our unit and only made a prescription of tenofovir disoproxil fumarate tablet 300 mg/day for antiviral therapy. By the 4th day of consecutive therapeutic PE, his platelet count rose to 78 × 10^9^/L and LDH level decreased to 730 U/L. However, the percentage of schistocytes on a peripheral blood smear rose to 30%, which was higher than the result on admission (Fig. 1). Meanwhile, the results of ADAMTS 13 activity and antibody were < 5% (68–131) and 181.34 ng/ml (131.25–646.5), respectively, which confirmed the diagnose of TTP. Other malignant and autoimmune diseases were also detected and found to be negative. PE was paused after 8 times therapy, and the LDH level was normal and platelet count remained over 150 for two consecutive days. At discharge on 15th day of admission, his CBC was as follows: Hb 107 g/L, hematocrit 34.1%, platelet count 195 × 10^9^/L, reticulocytosis of 6.55%, biochemical tests showed normal LDH and bilirubin levels. While the ADAMTS 13 activity and antibody were < 5% (68–131) and 272.22 ng/ml (131.25–646.5).

**Fig. 2:**
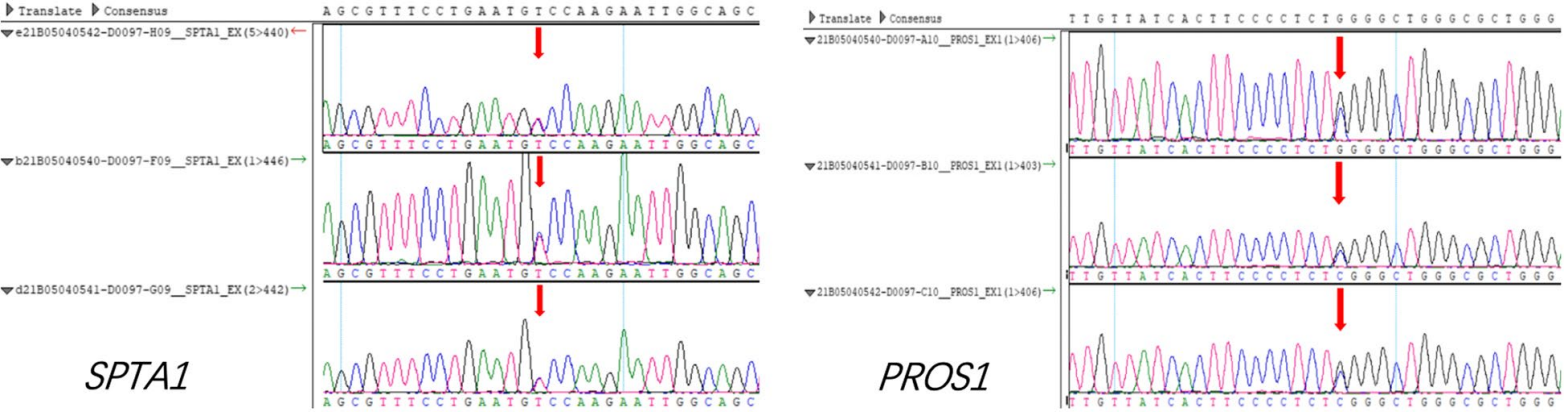
The genetic analysis revealed *SPTA1* and *PROS1* mutations

In order to distinguish the acquired autoimmune TTP and hereditary TTP, a genetic analysis was performed to analyze the *ADAMTS13* gene of the patient, his father (his mother was died by accident) and his son. Prior to the analysis we obtained informed consent from all of these individuals and received approval from the genetic ethics committee of our hospital. The analysis revealed that the patient’s father, himself and his son were heterozygous carriers of *SPTA1* and *PROS1* mutations instead of *ADAMTS 13* (Fig. 2).

Half a month after discharge, his laboratory evaluation revealed Hb level of 142 g/L, reticulocytosis of 1.82%, platelet count of 200 × 10^9^/L, LDH 174 U/L and 1.5% schistocytes on a peripheral blood smear. The ADAMTS 13 activity also elevated to 74.5%.

## Discussion and conclusions

TTP is a very rare, progressive, life-threatening form of thrombotic microangiopathy (TMA), which can be inherited or acquired. Thrombocytopenia, microangiopathic hemolytic anemia, fever, renal impairment and central nervous system involvement are the so-called TTP pentad [1]. However, TTP can present without the full pentad, up to 35% of patients do not have neurological signs at presentation and renal abnormalities or fever are not prominent features. The diagnosis of TTP is often based on clinical and laboratory finds, especially the test of ADAMTS13 activity and ADAMTS13 antibody. With the absence of ADAMTS13, leading to the accumulation of platelet-hyperadhesive and spontaneous formation of microthrombi within the microcirculation. ADAMTS13 deficiency may be due to either recessively inherited mutations of the *ADAMTS13* gene (hereditary TTP, also termed Upshaw–Schulman syndrome) or due to autoantibodies to ADAMTS13 (acquired autoimmune TTP). Certainly, the possibility of acquired autoimmune TTP cannot be completely ruled out, even if autoantibodies to ADAMTS13 are negative. Acquired TTP is a rare thrombotic microangiopathy with an incidence of approximately 2.2 cases per million per year. And, the inherited TTP represents less than 5% of all cases of TTP. In the absence of treatment, TTP is a rapidly fatal disease (mortality rate > 90%). During the past two decades, the introduction of therapeutic plasma exchange and plasma infusion has led to a decrease in the mortality rate to around 15%. The etiology of acquired TTP including HIV infection, pregnancy, autoimmune disease (SLE), organ transplantation, drugs (quinine, interferon, cyclosporine, ticlopidine and mitomycin C), malignancy and so on [2].

Interferon therapies are widely used for the treatment of neoplastic, autoimmune and infectious diseases, and the complications associated with interferon treatment include flu-like symptoms, headache, anaemia, thrombocytopenia, cardiovascular and gastrointestinal systems disorders. The two most common forms of TMA are TTP and haemolytic uremic syndrome (HUS), which can be resulted from interferon treatment among a few cases in multiple sclerosis, Sezary syndrome, chronic myelogenous leukaemia, hairy cell leukaemia and hepatitis C [3]. It is not certain whether this reflects a predisposition of haematological malignancies to cause TMA or is related to duration and dose of interferon therapy. Although different mechanisms have been proposed, the causative mechanism of interferon-induced TMA remains elusive. Kavanagh et al. provided the evidence of a causal link between interferon therapy and TMA based on clinical and experiments finds. They believed that drug-induced TMA could be caused by a direct toxic effect of the drug or via indirect immune-mediated mechanisms [4]. As to dose-dependent interferon-associated microangiopathy included endothelial hyperplasia, luminal occlusion and microaneursym formation. Haiyan et al. also demonstrated the detrimental effects of interferon on endothelial cell functions mediated with angiogenesis and fibrinolysis, which could potentially cause the loss of physiological endothelium thromboresistance and facilitate the development of vascular complications [5].

The development of vaccines to fight COVID-19 has been a remarkable medical achievement [6]. However, this widely immunization effort has been challenged by a rare vaccine-induced immune thrombotic thrombocytopenia (VITT). There are several reports show that no matter COVID-19 infection or COVID-19 vaccine injection can induce acquired TTP, which mostly occurred accompanying with the COVID-19 infection or usually within two weeks after vaccination. The exact pathophysiology of them remains to be clarified. While direct diffused endothelial cell injury plays a vital role, of course, cytokine storm, immune complex and low ADAMTS13 activity are also been reported to be associated with the pathophysiology of VITT [7].

In our case, we think the cause of TTP is interferon related instead of the VITT, because of the vaccine injection is almost one year ago, which is exclude by the recent guidelines based on expert opinions from the American Society of Hematology or the International Society on Thrombosis and Haemostasis [8]. Moreover, this patient got peginterferon-2 α 180 ug/week injection for nearly three years, which may meet the criteria of drug-dose dependent injury. And genetic analyses for the patient and his family in this case further confirm our diagnosis. Although we do not detect mutation of *ADAMTS13* gene, the *SPTA1* and *PROS1* mutations also remind us to alert the diseases of hereditary spherocytosis and protein S deficiency [9, 10].

For now, the diagnosis of TTP is still challenged by the rarity of the disease, which may delay the management of the tricky disease and affect the prognosis. The case of a young patient with acquired TTP is unusual because it gives us some lessons to recognize and distinguish the causes of TTP between pegylated interferon and COVID-19 vaccine injection. Elucidating the correct and early detection of ADAMTS13 deficiency is crucial for the patient’s prognosis. The correct diagnosis allows appropriate treatment, which is vital to prevent severe sequelae.

## Data Availability

All data collected from the patient were obtained from Changzheng Hospital and were available in this paper.

## Abbreviations

CBC: Complete blood count
Hb: Haemoglobin
LDH: Lactate dehydrogenase
TTP: Thrombotic thrombocytopenic purpura
PE: Plasma exchange
TMA: Thrombotic microangiopathy
HUS: Haemolytic uremic syndrome
VITT: Vaccine-induced immune thrombotic thrombocytopenia
